# Fungal osteomyelitis and septic arthritis in an immune competent man: The first report of invasive osteoarticular infection due to *Scedosporium dehoogii*

**DOI:** 10.1016/j.mmcr.2021.06.001

**Published:** 2021-06-19

**Authors:** Daniel S. Krauth, Brian T. Barlow, Catherine M. Berjohn

**Affiliations:** a Division of Infectious Diseases, Naval Medical Center, San Diego, 92134, United States; b Department of Medicine, Uniformed Services University of the Health Sciences, Bethesda, 20814, United States; c Department of Orthopaedic Surgery, Naval Medical Center, San Diego, 92134, United States; d Department of Surgery, Uniformed Services University of the Health Sciences, Bethesda, 20814, United States

**Keywords:** Scedosporium: dehoogii: invasive: Osteomyelitis: Septic arthritis

## Abstract

Invasive osteoarticular infections (IOI) due to Scedosporium spp. are rare in the immune competent patient, but have been associated with direct inoculation from antecedent trauma. Here we describe a case of IOI due to Scedosporium dehoogii in a previously healthy man. The clinical presentation and the diagnosis and treatment is discussed. To our knowledge, this is the first reported case of IOI caused by S. dehoogii.

## 1 Introduction

*Scedosporium* spp. are filamentous fungi found in soil and water worldwide [1]. The genus of this mold is defined as an anamorph, in comparison to its related teleomorphs within the *Pseudallescheria boydii* complex [2]. Indeed, taxonomy among *Scedosporium* spp. and related molds have become more carefully stratified through gene sequencing in addition to classical morphologic and physiologic characterization [3]. In turn, an expansion of fungal deoxyribonucleic acid (DNA) sequence databases has occurred and has facilitated more specific speciation of clinical *Scedosporium* spp. isolates [4]. One of such more recently characterized *Scedosporium* spp. is *S. dehoogii* which was initially characterized from soil and human skin in 2008 [3].

Clinically, scedosporiosis is the array of human disease caused by *Scedosporium* spp. The most common risk factors for scedosporiosis are severe immunosuppression, as well as structurally abnormal lungs where invasive disease is most commonly found [1]. Invasive osteoarticular infections (IOI) due to *Scedosporium* spp. are rare, but may occur in the immune competent patient after trauma with direct inoculation [5]. *S. dehoogii* identification from clinical isolates causing obvious disease is rare [6] and only described recently in three case reports. *S. dehoogii* was isolated from a child with endophthalmitis after penetrating eye trauma [7], in an immune compromised man with cutaneous disease [8], and also in an immune competent man with psoriasis who experienced a self-limited subcutaneous infection [9].

To date, *S. dehoogii* has not been described as a cause of deep-seated disease. Using ribosomal DNA sequencing technology, we describe the identification of *S. dehoogii* as cause for extensive IOI in a previously healthy man.

## 2 Case presentation

An exceptionally active 45 year-old-male activated United States (US) Army Reservist with no past medical history presented for review after sustaining a laceration to his right knee.

The patient was conducting marijuana abatement when he accidentally lacerated his right leg just proximal and lateral to the knee joint resulting in a superficial soft tissue injury. He was examined in the emergency department (ED) later that day (day 0). He received 10 sutures, tetanus immunization, and was prescribed cephalexin 500mg to be taken orally four times daily for seven days. He had excellent healing of the wound with rapid advancement in his mobility and remained without constitutional symptoms, associated erythema, or significant pain. On day +7 after the laceration, he fell down stairs onto his right knee. There was mild pain at that time with no popping or tearing sensation. However, over the next 72 hours, he noted increased pain and swelling of the right knee. Post-injury day +10 was also the first time he noticed an appreciable impairment in mobility due to pain. He had a second fall which worsened his knee pain further. The swelling and pain did not improve by day +35 so he presented again to the ED for reevaluation. His vital signs were normal and he was afebrile. His knee was warm, swollen, but without erythema. Range of motion was full, but painful with extremes of motion. Laboratory data revealed an elevated C-reactive protein (CRP) level of 5.4 mg/dL (reference <0.5 mg/dL), but other blood tests did not reveal any abnormalities. Arthrocentesis was advised, however, the patient declined the procedure. Meniscus injury was atop the differential diagnosis by the ED team and orthopedics due to his repeated falls; infection was believed to be unlikely due to the rapid initial healing and long temporal distance between injury and re-presentation to the ED. No antibiotics were prescribed and he was scheduled for magnetic resonance imaging (MRI) of the knee as an outpatient on day +49.

**Fig. 1:**
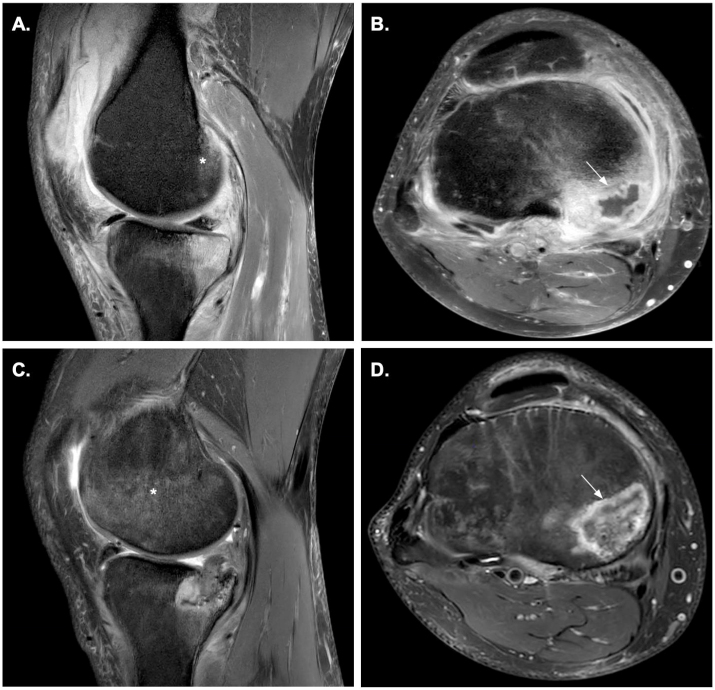
Magnetic resonance imaging (MRI) of the right knee with and without contrast acquired on day +49 before surgery (A. and B.), and day +195 after initial debridement in the joint space and the medial tibial plateau (B. and C.). Fat suppressed proton density (FS-PD) MRI sequences demonstrating osteomyelitis within the lateral femoral condyle (Labeled with “*”; A. and C.). T-2 weighted MRI sequences demonstrating medial tibial plateau osteomyelitis with abscess 2.3 × 1.3 × 0.3cm, and post-debridement changes (Labeled with arrows; B. and D., respectively).

Right knee MRI with and without contrast revealed findings concerning for osteomyelitis within the lateral femoral condyle (Fig. 1A) and the medial tibial plateau osteomyelitis with abscess formation (2.3 × 1.3 × 0.3cm; Fig. 1B). A joint effusion and thickened enhancing synovium was present consistent with septic arthritis. The MRI also demonstrated enhancement of the popliteus musculature compatible with myositis and a longitudinal tear of the medial meniscus. The patient underwent urgent arthroscopic washout and debridement on day +50. He was initially treated empirically with intravenous ceftriaxone and vancomycin. Numerous serum based microbiologic diagnostics were negative and bone cultures from the tibial sequestrum yielded no growth. Pathologic analysis of bone revealed lymphoplasmacytic necrosis and osteomyelitis without the identification of bacterial, mycobacterial, or fungal elements. Due to concerns for difficult-to-grow bacteria, mycobacteria, or fungi as cause of infection, bone biopsy under fluoroscopic guidance was then pursued on day +57 for repeat biopsy of the tibial plateau sequestrum and sample processing for direct molecular based testing. At discharge the patient was receiving oral linezolid, rifampin, and moxifloxacin as empiric coverage for most bacterial pathogens and non-tuberculous mycobacteria. Universal broad-range 28S ribosomal polymerase chain reaction (uPCR) assay revealed the presence of *Scedosporium dehoogii* DNA. The patient's antimicrobials were then transitioned to dual antifungal therapy with oral voriconazole (VCZ) and oral terbinafine (TBF). He first received two 400mg loading doses of oral VCZ 12 hours apart, followed by oral voriconazole 300mg twice daily. VCZ levels were acquired every 14 days with a target dosing range between 2 and 6 mg/L. Oral TBF was dosed 250mg once daily.

**Fig. 2:**
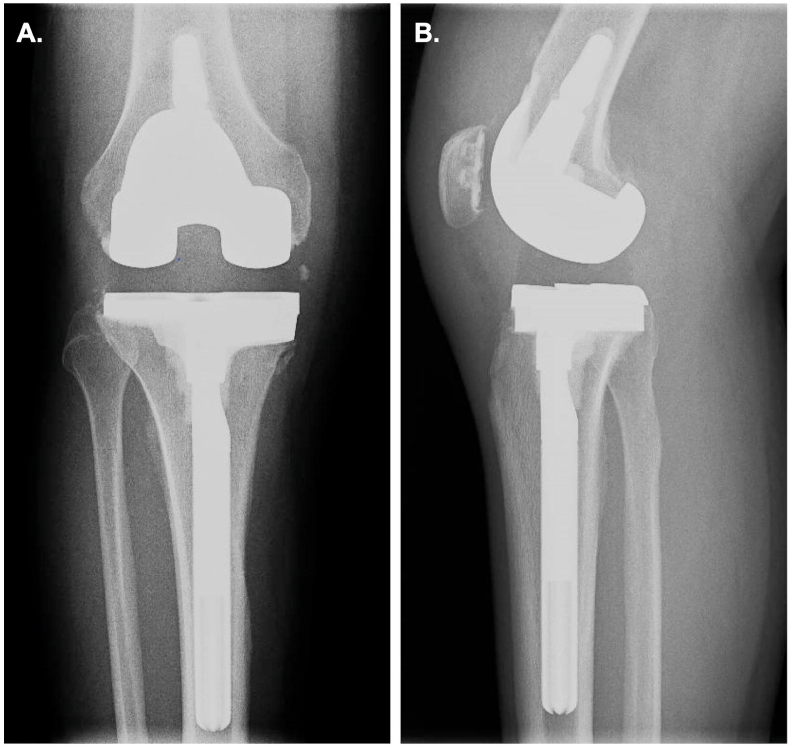
Anterior (A.) and lateral (B.) weight bearing X-ray films of the right knee on day +621 after revision of Zimmer Persona total knee arthroplasty (TKA) was performed on day +561.

While maintained on VCZ and TBF, his CRP normalized and average VCZ levels were 4 mg/L for the next several months. Repeat MRI on day +195 revealed ongoing osteomyelitis in the lateral femoral condyle and extensive post-debridement changes in the medial tibial plateau (Fig. 1C and D). He underwent a repeat arthroscopic debridement on day +242 for continued severe debilitating knee pain and continued radiographic advancement of disease in the tibia, but unfortunately the procedure did not successfully improve his symptoms. He had requested an amputation due to the severe debilitating pain despite appropriate antifungal treatment and multiple attempts at arthroscopic debridement. He was indicated for a two stage total knee arthroplasty (TKA). He had an en bloc resection of the affected osteomyelitic medial tibial plateau and an articulating VCZ spacer with a 15mm medial augment was placed on day +463. The resected bone from the medial tibia was sent for fungal cultures and uPCR after the first stage of knee arthroplasty and remained negative. Dual antifungal therapy was uninterrupted until he was placed on a 4 week antifungal holiday starting on day +533 with continued demonstration of low level inflammatory assays. He underwent a second stage reconstruction on injury day +561 with a revision Zimmer Persona TKA. The day after completion of the surgery, the patient was restarted on VCZ and TBF at the same previous doses, including the same VCZ loading dose regimen as when he first started antifungals. On evaluation of day +576, the patient confirmed appreciable reduction in his knee pain with no infection complications identified. Follow-up X-rays of the patient's right knee on day +621 confirmed appropriately maintained hardware alignment (Fig. 2A and B). Long term VCZ and TBF for at least six months after his second stage TKA was recommended despite fingernail discoloration and peripheral neuropathy symptoms to reduce his risk of treatment failure.

## 3 Discussion

This case represents the first confirmed IOI scedosporiosis due to *S. dehoogii* in the English-language literature. Pathogenesis began after direct inoculation from an outdoor trauma which parallels other cases of IOI due to other *Scedosporium* spp. [5]. While *S. dehoogii* has been isolated from various clinical specimens, it has only been reported as the cause of human disease in three cases [[7], [8], [9]]. Environmental isolates of *S. dehoogii* have been found to cause significantly higher rates of death in a mouse model after inoculation compared other *Scedosporium* spp. [10], however, virulence in humansremains poorly understood.

The identification of *S. dehoogii* in this case was achieved by broad-range 28S ribosomal PCR sequencing directly upon infected bone. Various fungal sequencing platforms allow for the detailed speciation of *Scedosporium* spp. [4,6]. and the use of multiplex PCR assays upon clinical isolates to identify molds can be invaluable when acquired cultures are negative [11]. While sensitivity of uPCR may be limited, diagnostic yield is highest when performed upon tissue [11]. If the specific *Scedosporium* spp. cannot be isolated for direct antifungal susceptibility testing (AST), identification of the specific species may assist in both prognostication [12] as well as guiding antifungal therapy [13].

A systematic review of non-*Aspergillus* related IOI revealed that *Scedosporium* spp. (not including *Lomentospora prolificans*) comprises one-third of all fungal IOI, after *Fusarium* spp. [14]. The management of non-*Aspergillus* IOI most often includes both surgical debridement and antifungal therapy and results in variable cure rates and outcomes [6,14,15].

*S. dehoogii* in particular is extraordinarily difficult to treat. Unlike other members of its genus, it is poorly susceptible to most antifungal therapy.

Medically, VCZ is the first-line therapy for invasive scedosporiosis [[15], [16], [17]] and treatment duration is usually months. VCZ has excellent bone penetration, but appears to concentrate poorly in the synovium [18]. Regarding *S. dehoogii*, there is a paucity of data regarding optimal therapy and if AST can be performed on an isolate, synergy testing should also be considered.

An analysis of species-specific antifungal susceptibilities was conducted upon clinical and environmental isolates, including *S. dehoogii*. Three clinical isolates, one from a patient with cystic fibrosis and two from skin, and 19 environmental isolates of *S. dehoogii* demonstrated minimum inhibitory concentrations (MIC) of 8 μg/mL against VCZ, and MICs of >16 μg/mL against amphotericin B, caspofungin, micafungin, itraconazole, isavuconazole, and posaconazole among of 90% of the isolates (MIC_90_; 14). Surgical intervention and topical/oral VCZ was successfully used to treat endophthalmitis caused by *S. dehoogii* in clinical practice [7]. In an iatrogenically immunosuppressed patient with cutaneous *S. dehoogii* disease, oral VCZ with local hyperthermia was used for two weeks (due to drug induced liver toxicity), followed by a year of oral itraconazole and TBF (MICs 0.25, 0.25, and >4 μg/mL, respectively) with eventual resolution [8].

Our approach to antifungal therapy, without the ability to perform AST, was based on published laboratory [13] and pathogenic clinical isolates of *S. dehoogii* [[7], [8], [9]], in addition to guidelines for the treatment of invasive scedosporiosis [16]. The addition of TBF to VCZ was based on the potential for synergy [1,19] and improved clinical outcomes in the extraordinarily difficult to treat *L. prolificans* [20]. In our patient, he continues to rehabilitate well after arthroplasty, and has been tolerating a prolonged course of antifungals.

In conclusion, this is the first confirmed case of IOI due to *S. dehoogii*. Diagnosis was achieved here by uPCR, and when traditional diagnostics are unable to identify a pathogen, infected tissue should be considered for uPCR analysis. The treatment of *S. dehoogii*, as well as other *Scedosporium* spp., often rests heavily on combined procedural source control, and first-line medical therapy is with VCZ. Additional antifungals, such as TBF, should be considered for synergy.

## Declaration of competing interest

None to report.
